# Drug Discovery for Schistosomiasis: Hit and Lead Compounds Identified in a Library of Known Drugs by Medium-Throughput Phenotypic Screening

**DOI:** 10.1371/journal.pntd.0000478

**Published:** 2009-07-14

**Authors:** Maha-Hamadien Abdulla, Debbie S. Ruelas, Brian Wolff, June Snedecor, Kee-Chong Lim, Fengyun Xu, Adam R. Renslo, Janice Williams, James H. McKerrow, Conor R. Caffrey

**Affiliations:** 1 Sandler Center for Basic Research in Parasitic Diseases, California Institute for Quantitative Biosciences (QB3), University of California, San Francisco, California, United States of America; 2 Small Molecule Discovery Center, California Institute for Quantitative Biosciences (QB3), University of California, San Francisco, California, United States of America; McGill University, Canada

## Abstract

**Background:**

Praziquantel (PZQ) is the only widely available drug to treat schistosomiasis. Given the potential for drug resistance, it is prudent to search for novel therapeutics. Identification of anti-schistosomal chemicals has traditionally relied on phenotypic (whole organism) screening with adult worms *in vitro* and/or animal models of disease—tools that limit automation and throughput with modern microtiter plate-formatted compound libraries.

**Methods:**

A partially automated, three-component phenotypic screen workflow is presented that utilizes at its apex the schistosomular stage of the parasite adapted to a 96-well plate format with a throughput of 640 compounds per month. Hits that arise are subsequently screened *in vitro* against adult parasites and finally for efficacy in a murine model of disease. Two GO/NO GO criteria filters in the workflow prioritize hit compounds for tests in the animal disease model in accordance with a target drug profile that demands short-course oral therapy. The screen workflow was inaugurated with 2,160 chemically diverse natural and synthetic compounds, of which 821 are drugs already approved for human use. This affords a unique starting point to ‘reposition’ (re-profile) drugs as anti-schistosomals with potential savings in development timelines and costs.

**Findings:**

Multiple and dynamic phenotypes could be categorized for schistosomula and adults *in vitro*, and a diverse set of ‘hit’ drugs and chemistries were identified, including anti-schistosomals, anthelmintics, antibiotics, and neuromodulators. Of those hits prioritized for tests in the animal disease model, a number of leads were identified, one of which compares reasonably well with PZQ in significantly decreasing worm and egg burdens, and disease-associated pathology. Data arising from the three components of the screen are posted online as a community resource.

**Conclusions:**

To accelerate the identification of novel anti-schistosomals, we have developed a partially automated screen workflow that interfaces schistosomula with microtiter plate-formatted compound libraries. The workflow has identified various compounds and drugs as hits *in vitro* and leads, with the prescribed oral efficacy, *in vivo*. Efforts to improve throughput, automation, and rigor of the screening workflow are ongoing.

## Author Summary

The flatworm disease schistosomiasis infects over 200 million people with just one drug (praziquantel) available—a concern should drug resistance develop. Present drug discovery approaches for schistosomiasis are slow and not conducive to automation in a high-throughput format. Therefore, we designed a three-component screen workflow that positions the larval (schistosomulum) stage of *S. mansoni* at its apex followed by screens of adults in culture and, finally, efficacy tests in infected mice. Schistosomula are small enough and available in sufficient numbers to interface with automated liquid handling systems and prosecute thousands of compounds in short time frames. We inaugurated the workflow with a 2,160 compound library that includes known drugs in order to cost effectively ‘re-position’ drugs as new therapies for schistosomiasis and/or identify compounds that could be modified to that end. We identify a variety of ‘hit’ compounds (antibiotics, psychoactives, antiparasitics, etc.) that produce behavioral responses (phenotypes) in schistosomula and adults. Tests in infected mice of the most promising hits identified a number of ‘leads,’ one of which compares reasonably well with praziquantel in killing worms, decreasing egg production by the parasite, and ameliorating disease pathology. Efforts continue to more fully automate the workflow. All screen data are posted online as a drug discovery resource.

## Introduction

Treatment and control of the flatworm disease, schistosomiasis, relies on a single drug, praziquantel (PZQ). Since the first clinical trials in the late 1970's [1], PZQ has proven safe and effective against all three major forms of the disease, and today, declining costs make the drug more affordable, currently at around 7–19 US cents per 600 mg tablet [2]. A single oral dose of 40–60 mg/kg is sufficient to achieve cure rates of 60–90% [3] while facilitating patient compliance, especially among children. Clinically relevant and widespread resistance, despite occasional and isolated incidences [4], has yet to occur. This fortuitous situation stands in contrast to the situation for some other ‘neglected tropical diseases’, (NTDs; [5]) for which antiquated and often toxic drugs must be parenterally administered over a number of days or weeks and which increasingly have problems associated with drug resistance [6]. Thankfully, concerted pharmaceutical discovery efforts via ‘public-private partnership’ (PPP) consortia [7],[8] are ongoing to address this desperate situation and robust ‘drug pipelines’ have been established which, hopefully, should yield new therapies over the next ten to 15 years. All of this recent activity has bypassed schistosomiasis, due in part to the tremendous success of PZQ.

Yet, reliance on a single drug to treat a population of over 200 million people infected and over 700 million people at risk over three continents [9] seems particularly perilous when considering the threat of drug resistance. Also, PZQ is not without problems. Principal among these is its relative inactivity against migratory juvenile and sub-adult worms [10],[11] meaning that, for effective treatment and sustainable control, PZQ must be given on a regular basis. Thus, recent discussions, as part of treatment landscape for human helminthiases in general [12], have focused on reawakening the need to search for alternatives to PZQ, including the development of combinations of drugs incorporating PZQ [13],[14]. The latter option, if more difficult and costly to develop, has the longer term benefit of extending the availability of PZQ while hindering the onset of resistance to this most valuable of drugs.

Only to a limited extent has the underlying rationale for inquiry of anti-schistosomal compounds (and anthelmintics in general) involved detailed knowledge of the molecular drug target or mechanism of action although there are some notable advances, e.g., inhibition of redox [15] and proteolytic enzymes [16], and heme aggregation [17]. The relative lack of validated molecular drug targets for this parasite is in stark contrast to those underpinning entire drug development portfolios of PPPs tackling other infectious diseases of global import such as malaria and the trypanosomiases [7],[8]. Hopefully, this paucity of targets can be better addressed with the recent availability of the draft genomes of both *Schistosoma mansoni* [18] and *S. japonicum*, and the first attempts to prioritize those targets ([19]; TDR Drug Targets Prioritization Database [20]). More common in schistosome drug discovery has been the complementary approach of phenotypic (whole organism) screening *in vitro* (usually with adult worms) and/or animal models of disease to measure compound efficacy [21]. These strategies are usually without specific knowledge of the target and/or mechanism of action (e.g., [22],[23]), or for which bioactivity has been characterized in other parasitological or biomedical settings [24],[25],[26]. They are of proven value. For example, PZQ was first developed as a veterinary cestocide before being tested in an animal model of schistosomiasis [27] and long before data regarding its mechanism of action was gathered. However, the pace of discovery with these techniques is somewhat slow, relying on a small number of research groups expert in handling the complex schistosome life cycle and working with both finite yields of parasite (adult parasites must be harvested from mammalian hosts) and long screen timelines (it takes approximately 30 days for *S. mansoni* infections to become patent in the mouse model [28]).

Here, we have taken an alternative approach to phenotypic screening by designing a three-component screen workflow built upon juvenile parasites (schistosomula) that are easily obtainable from the vector snails and in far greater numbers than adult parasites. The screen is formatted to 96-well microtiter plates thus providing increased throughput and improved interfacing with similarly formatted small molecule libraries maintained in-house at the UCSF Small Molecule Discovery Center (SMDC; http://smdc.ucsf.edu/). Adult parasite screens *in vitro* form the second component of the workflow that is completed with compound efficacy tests in a murine model of patent schistosomiasis. Two GO/NO GO filter points are strategically placed in the screen workflow to prioritize compounds more likely to meet the target product profile (TPP) for treatment of schistosomiasis and its demand for short course oral chemotherapy [29]. As constructed, the entire process is intended to streamline and accelerate the identification of hit compounds and chemistries *in vitro*, and leads *in vivo*.

The screen workflow was inaugurated using a library of commercially available and chemically diverse compounds. Approximately 41% of the library comprises drugs already approved for human use thereby opening the possibility for repositioning (re-profiling or re-purposing) [30] chemical entities as novel anti-schistosomals. The same collections have already provided a number of leads against other parasites [31],[32],[33]. Drug repositioning offers shortened development timelines and decreased risk with compounds having already passed regulatory clinical trials with full toxicological and pharmacokinetic profiles [7],[30]. All of this adds to up to significant potential cost savings –important in the context of diseases afflicting the poor for which investment returns will be marginal. The results accrued from the inaugural screen are promising in that a number of potent anti-schistosomal single compounds and chemical classes have been identified *in vitro*, some of which elicit demonstrable anti-schistosomal effects in the murine model of disease. Importantly, these and future data arising from the screen workflow are made public via various online portals to allow those interested examine and mine the outputs and, hopefully, identify their own opportunities for NTD drug development.

## Methods

### Maintenance of the *S. mansoni* life cycle

A Puerto Rican isolate is maintained in the laboratory using the intermediate snail host, *Biomphalaria glabrata* and the Golden Syrian hamster *Mesocricetus auratus* (5–6 weeks old; Simonsen labs) as the definitive host. Animals were maintained and experiments carried out in accordance with protocols approved by the Institutional Animal Care and Use Committee (IACUC) at UCSF. Infections with *S. mansoni* are initiated by subcutaneous injections of 800–1000 cercariae. At 6–7 weeks post-infection (p.i.), hamsters are euthanized with peritoneal (i.p.) injections of 50 mg/kg sodium pentobarbital and adult worms harvested by reverse perfusion of the hepatic portal system [34] in RPMI 1640 medium (Invitrogen, Carlsbad, CA).

### Preparation of schistosomula and adult parasites for the screen workflow

Upon exposure to light, 50–100 snails that are patent with *S. mansoni* infection, are induced to shed cercariae into the surrounding water. Cercariae are cleaned and concentrated over a series of sieves using distilled water and allowed to stand on ice in a 50 mL polystyrene tube for 1 h. During this time, cercariae clump, settle to the bottom and stick to the inside surface of the tube. The water is poured off and replaced with 9 mL ice-cold ‘Incomplete’ Medium 169 ([35]; custom made at the UCSF Cell Culture Facility) that contains 1× penicillin-streptomycin solution. Cercariae are mechanically transformed into schistosomula by passing back and forth between two 10 mL syringes attached via a 22-gauge double-headed needle (adapted from [36]). After deposition into a 9 cm diameter Petri dish, cercarial heads are separated from tails by swirling in Incomplete Medium 169 and the lighter tails aspirated leaving the heads (schistosomula) settled in the center of the dish. Under sterile conditions, schistosomula are washed 3 times in Incomplete Medium 169 and allowed to settle over ice in a 1.5 mL microfuge tube. Parasites are kept on ice for up to 2 h prior to screening with compounds. As a note, Medium 169 is preferred over RPMI as a culture medium for schistosomula – worms survive with <10% mortality for up to 4 weeks whereas in RPMI, approximately 40–60% of the parasites die within 3 days with continued mortality out to two weeks (Ruelas and Caffrey, unpublished).

Adult worms, perfused from hamsters, are washed 5 times in RPMI 1640 containing 1× penicillin-streptomycin solution and 10 µg/mL amphotericin B (both supplied by the UCSF cell culture facility). After 3 further washes in Incomplete Medium 169, parasites are maintained in ‘Complete’ Medium 169 (with the addition of 10% fetal bovine serum (FBS; HyClone, Logan, Utah) at 37°C and 5% CO_2_ for up to 24 h prior to screening with compounds.

### Compound storage and handling for the schistosomula component of the screen workflow

The ‘Spectrum’ and ‘Killer’ compound collections, together comprising 2,160 (1,992 unique) compounds were purchased from Microsource Discovery Systems, Inc. (Gaylordsville, CT, USA; http://www.msdiscovery.com/). Information on both is available for download as .xls files from http://www.msdiscovery.com/spectrum.html and http://www.msdiscovery.com/killer.html, respectively. Together the library contains synthetic compounds, natural products and drugs of which 821 are FDA-approved [31]. The library is maintained as 1 and 5 mM stocks in 384-well plates and −80 C at the UCSF Small Molecule Discovery Center that is juxtaposed to the UCSF Sandler Center.

**Figure 1:**
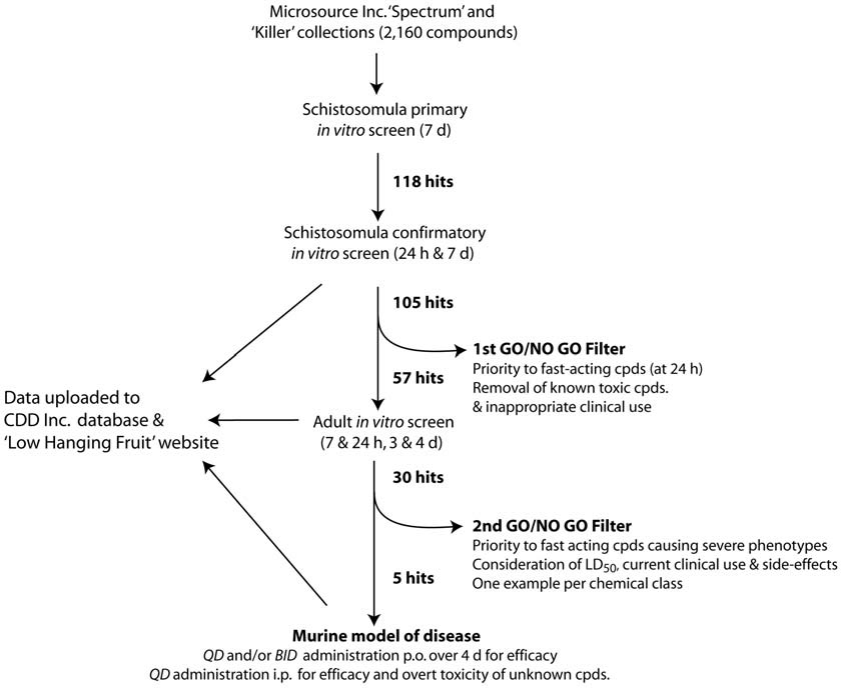
Workflow for phenotypic screening of *S. mansoni*. The workflow was prosecuted with the Microsource Discovery Inc.'s “Spectrum” and “Killer” collections that together comprise 2,160 (1,992 unique) compounds, including 821 drugs approved for use with humans (http://www.msdiscovery.com/). The goal was to interface this parasite with the 96-well plate-formatted small molecule libraries available at the UCSF Small Molecule Discovery Center (SMDC; http://smdc.ucsf.edu/) and common elsewhere, thereby accelerating throughput and facilitating screen automation. Schistosomula are placed at the apex of a three-component workflow that subsequently incorporates screens against adult parasites *in vitro* and finally an animal model of infection to measure *in vivo* efficacy. Times at which phenotypes were recorded *in vitro* are indicated in hours and days Two GO/NO GO workflow filters allow for prioritization of the ‘hit’ compounds *in vitro*. The numbers of ‘hits’ generated at various points in the workflow are indicated in bold typeface. Data arising from each of the three screening components are posted online as a flat file at The Sandler Center's ‘Low Hanging Fruit” website (http://pathology.ucsf.edu/mckerrow//fruit.html), and in a cross-searchable format, at the database maintained by Collaborative Drug Discovery (CDD Inc.; (http://www.collaborativedrug.com/). For smaller numbers of compounds, the workflow need not be hierarchically prosecuted, rather every compound can be screened against both schistosomula and adults.

For the first component of the screen workflow (see Figure 1 for schematic) involving primary screens of schistosomula, 96-well polystyrene dilution plates (Corning, MA) are prepared using a Matrix WellMate bulk dispenser and a Biomek FX^p^ liquid handling system. To these plates, the FX^p^ transfers 4 µL of 1 mM compound in neat DMSO from quadrants of the 384 well stock plates. Eighty compounds from each quadrant are transferred to each dilution plate leaving the outer two columns empty. The WellMate then dispenses 16 µL DMSO and 180 µL Incomplete Medium 169 to the dilution plates to yield 20 µM compound in 200 µL 10% DMSO. Finally, the FX^p^ transfers 10 µL of diluted compounds to the 96-well screen plates followed by 180 µL of Complete Medium 169. Under sterile conditions, 10 µL of schistosomula (200–300 worms) maintained on ice are added manually so that the final concentrations of test compound and DMSO per well are 1 µM and 0.5%, respectively. The outer two columns (1 and 12) of each screen plate are kept empty for eventual manual addition of the anti-schistosomal compounds, PZQ and the cysteine protease inhibitor, K777 [16], each at 1 and 5 µM. Plates are maintained at 37°C in a 5% CO_2_ atmosphere.

For confirmatory screens of schistosomula (Figure 1), consensus hits (details below) from the primary screen are ‘cherry picked’ using the Matrix WellMate and Biomek FX^p^. A custom protocol in Pipeline Pilot software (Accelrys, CA) generates an Excel file that is read by the FX^p^. This file is designed to randomly distribute hit compounds among wells containing only DMSO (‘dummy wells’) in the dilution plate. To start the liquid-handling procedure, the WellMate transfers 48 µL of neat DMSO into the inner 80 wells of 96-well polystyrene dilution plates. From 5 mM stocks in neat DMSO, the Span 8 arm on the FX^p^ then transfers 2 µL of consensus hit compounds (or DMSO from a plate containing 100% DMSO) into the dilution plates. To complete the dilution plates, 150 µL of Incomplete Medium 169 are added to each well. From this dilution plate, 4 µL are transferred into the 96-well screen plates followed by 186 µL of Complete Medium 169. Under sterile conditions, 10 µL schistosomula (200–300) are then added manually to yield final concentrations of 1 µM and 0.5% for test compound and DMSO, respectively.

### Compound handling for the adult component of the screen workflow

For the second component of the screen workflow involving screening of adult *S. mansoni* (Figure 1), the FX^p^ Span 8 transfers 4 µL 5 mM hit compounds in neat DMSO into 96-well polystyrene dilution plates. The hits are distributed randomly in the first few rows of these plates, but with fewer dummy DMSO wells to accommodate the smaller 24-well screen plates. Then, 96 µL of neat DMSO are added to this plate using the WellMate bulk dispenser. From this, 10 µL of the diluted compounds are added manually to the 24-well screen plates and immediately mixed with 0.99 mL of Complete Medium 169 to prevent evaporation of DMSO. Under sterile conditions, adult worms (4–8 pairs) are manually added in 1 mL Complete Medium 169. Final concentrations of test compound and DMSO are 1 µM and 0.5%, respectively.

### Phenotype scoring and compound concentration

Two screen analysts spent approximately four weeks testing the Microsource ‘Killer Collection’ with both schistosomula and adult parasites in order to familiarize themselves with the types of phenotypes arising and their changes as a function of time. Phenotypes were scored using a Zeiss Axiovert 40 C inverted microscope and ×10 and ×2.5 objective lens for schistosomula and adults, respectively.

Screening analysts are blind to the compound identities which are not disclosed until the conclusion of each of the schistosomular and adult components of the workflow. As a further precaution against subjective bias, each screen analyst visually scores and characterizes phenotype ‘hits’ in isolation. Both analysts then compile ‘consensus hits’. In those cases for which a consensus cannot be reached the compounds in question are scheduled for re-screening. With repetition, the failure rate to identify consensus hits was decreased to less than 5% per plate for both the schistosomula and adult components of the workflow.

It was also during the four week training period that a decision was reached on the compound concentration at which the Microsource collections would be screened. Initial testing of the Killer collection at 10 and 5 µM with schistosomula yielded too many hits (average of 25 and 15% of the 80 compounds per plate, respectively) to subsequently perform, in a reasonable time-frame, *in vitro* screening with the more limiting adult parasites. At 1 µM, however, an average 10% hit rate was achieved.

For primary screens of schistosomula, phenotypes were monitored after 7 d a time frame considered long enough to record the development of any potentially relevant phenotype (Figure 1). For confirmatory screens with schistosomules, phenotypes were scored after 24 h and 7 days in order to identity fast-acting compounds and re-confirm the data from the primary screen, respectively. For adult screens, phenotypes were monitored after 7 and 24 h, and, thereafter, daily up to 4 days (Figure 1).

### GO/NO GO filters in the screen workflow

Two GO/NO GO filters are positioned in the screen workflow in order to prioritize which compounds go forward (Figure 1) based upon the TPP for schistosomiasis treatment and its demand for short course oral therapy [29].

The first filter, placed between the schistosomular and adult components of the workflow, prioritizes compounds yielding phenotypes by 24 h and removes compounds (where data are available) that are clearly toxic and/or unsuitable for oral administration. The second GO/NO-GO filter, upon completion of the adult screen component, prioritizes those hit compounds for tests in the murine model of schistosomiasis mansoni. This prioritization is more complex than the first filter as a number of parameters must be simultaneously considered. Primary emphasis is placed on the time to appearance of the phenotype plus the severity of that phenotype, e.g., fast-acting ‘death’ phenotypes (<24 h) are most preferred. Other factors influencing the decision include clinical indication (if known) for the compound including undesirable side-effects (e.g., hormones disallowed; psychoactives less preferred); oral bioavailability (preferred over other routes of administration) and data on acute toxicity (e.g., LD_50_ oral (p.o.), i.p., and/or intra-venous (i.v.)). Finally, where compounds with similar chemistries are represented more than once, a single example is initially considered for tests in the mouse model.

### Murine model of schistosomiasis mansoni

The third and final component of the *S. mansoni* screen workflow (Figure 1) entails infections of 4–6 week-old Swiss Webster mice (Simonsen Laboratories) with *S. mansoni* that are initiated by subcutaneous injection of 140 cercariae. Experiments were carried out in accordance with protocols approved by the Institutional Animal Care and Use Committee (IACUC) at UCSF. Groups of 4 or 5 mice are used per treatment. Commencing on day 42 p.i. when *S. mansoni* infections are patent (i.e., when parasite eggs are present in feces) compound is administered once daily (QD) and/or twice daily (BID) for 4 days. This time period is considered sufficient to record any compound efficacy given that the desired TPP for any new-anti-schistosomal calls for short course therapy [15],[29]. Compound is administered p.o. (vehicle is 2.5% Cremophor EL unless otherwise stated) or, i.p., when data on oral bioavailability are not to hand. The amount of compound to administer is guided by available LD_50_ values for acute toxicity, in which case compound is given close to that value in order to determine whether a therapeutic window exists. Overt toxicity of compounds (e.g., death and behavioral changes) is assessed daily during and after treatment until the time of euthanasia. At 55 days p.i., mice are euthanized with an i.p. injection of 0.05 mg/g sodium pentobarbital, and adult worms perfused as described above for hamsters.

Compound efficacy *in vivo* is measured as described [16] using a number of criteria and is compared to that of the anti-schistosomal drug, PZQ, as a ‘gold standard.’ The criteria include the parasitological parameters of numbers of male and female worms recovered by perfusion, and hepatic egg burdens. Also, the amelioration of pathology as evidenced by decreased liver and spleen weights is recorded. Attention is also paid to worm size upon recovery. To recover eggs trapped in liver, whole livers (or the caudate liver lobe, see below) from individual mice are excised, weighed and digested in 0.7% porcine trypsin in PBS for 1 h at 37°C on an orbital shaker. Eggs are sedimented at 4°C and counted under a dissecting microscope as described previously [37].

### Statistics

Data for worm and hepatic egg counts, and organ weights, were compiled on a per mouse basis and median values calculated per treatment group. All data were subjected to the Mann-Whitney nonparametric test to determine any statistical differences in egg and worm burdens, and organ pathologies between treated and untreated control mice. As an expedient alternative in some experiments, particularly in those cases where worm burdens were not dramatically decreased, hepatic egg counts were calculated as an average per treatment group rather than per mouse. To do this, the caudate liver lobe from individual mice was excised, pooled per treatment group and weighed prior to trypsin digestion and counting of eggs. The single value arising was then calculated with respect to the total liver weight in the group and then divided by the number of mice in the group.

## Results

### Phenotype classification for schistosomula

**Figure 2:**
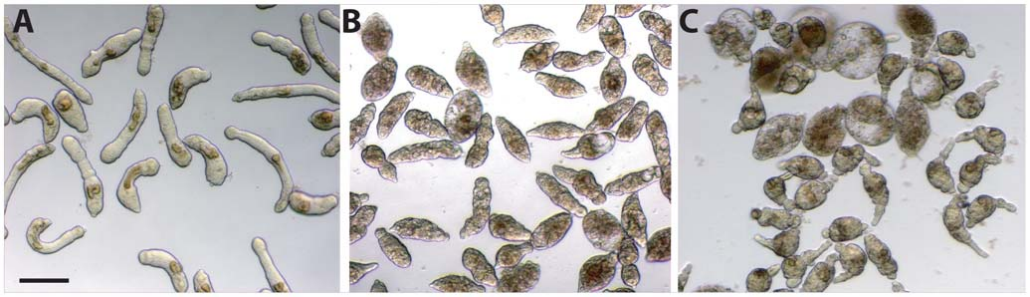
Examples of the different and multiple phenotypes manifest by schistosomula exposed to chemical insult. Worms were exposed for 7 days to 1 µM of either the schistosomicidal cysteine protease inhibitor, K777 (B), or the current chemotherapy, PZQ (C), and compared to controls with DMSO alone (A). Phenotypes ascribed are ‘dark/dead’ for K777 and ‘overactive/degenerated but mobile’ for PZQ. Images were captured using a Zeiss Axiovert 40 C inverted microscope (10× objective) and a Zeiss AxioCam MRc digital camera controlled by AxioVision 40 version 4.5.0.0 software. Scale bar = 0.2 mm.

During initial testing of the Microsource ‘Killer Collection’, it became clear that schistosomula display different and often multiple phenotypes that change over time. Eventually, we could consistently ascribe six phenotypes to worms under chemical insult relative to control worms exposed to 0.5% DMSO (Figure 2; Table S1). The phenotype terms we employed range from the obvious (‘dead’, ‘overactive’, and ‘rounded’) to the more sublime (‘dark’, ‘slow’), yet, nonetheless, clearly distinguishable from DMSO controls. An example of the overactive phenotype is shown in Video S1 which should be compared with normal worm movement displayed in Video S2. The last phenotype, ‘degenerate but mobile’, describes those cases in which the worms are clearly motile yet severely disrupted in morphology. An example of this phenotype is produced by PZQ, used as a ‘gold standard’ schistosomicide throughout the screen workflow. PZQ initially elicited an overactive phenotype (observed within 10 mins) that progressed to a combination of ‘overactive/degenerate but mobile’ by 7 days (Figure 2C). In contrast, the cysteine protease inhibitor, K777 [16], also used as a standard compound, had a more progressive effect; “slow/dark” by 3 days leading to ‘dark/slow/dead’ by 7 days (Figure 2B).

### Phenotype classification for adult worms

**Figure 3:**
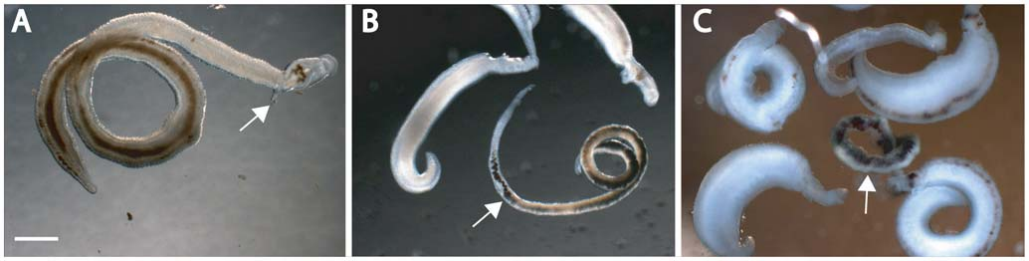
Examples of the different and multiple phenotypes manifest by adult *S. mansoni* exposed to chemical insult. Worms were exposed for 2 days to 1 µM of either K777 (B) or PZQ (C) and compared to controls with DMSO alone (A). Phenotypes ascribed are ‘slow/sex separated/on sides’ for K777 and ‘slow/dark/shrunken/on sides/sex separated/tegumental blebbing’ for PZQ. Arrows point to female worms. Images captured using a Zeiss 2000-C Stemi inverted microscope mounted over a Diagnostic Instruments Transmitted Light Base and a Zeiss AxioCam MRc digital camera controlled by AxioVision 40 version 4.5.0.0 software. Scale bar = 0.7 mm.

Similar to schistosomula, adult worms could manifest multiple and changing phenotypes in response to chemical insult (Figure 3; Table S2). There was some overlap in the phenotypes classified compared to schistosomula: ‘dead’, ‘dark’, ‘slow’ and ‘overactive’ remained relevant whereas ‘rounded’ and ‘degenerate but mobile’ did not. Additional, adult-specific phenotypes were: ‘tegumental blebbing’ (teg. bleb.) to document damage to the surface (tegument) of the adult; ‘sexes separated’ (sex sep.), whereby the male and female worms become unpaired, and the self-evident ‘shrunken.’ For example, PZQ, elicited ‘shrunken/dark/slow’ phenotypes (observed within 2 min of addition of 1 µM PZQ) that progressed to ‘shrunken/dark/sexes sep/teg. bleb/dead’, by 4 days (Figure 3C). In contrast, K777 had a more progressive effect; ‘slow/dark’ by 2 days leading to ‘dark/slow/sexes sep/on sides/dead’ by 4 days (Figure 3B).

### Schistosomula primary and confirmatory screens

Of the 1,992 unique compounds comprising the Microsource Spectrum and Killer collections, 118 yielded phenotypes (termed ‘hits’ representing 5.9% of the total) in the schistosomula primary screen component of the workflow after 7 days at a concentration of 1 µM. The compound names, structures, therapeutic uses and phenotypes identified are listed in Table S1. The majority of these (105) were returned as hits in the confirmatory schistosomula screen component after 7 days and, of these, 61 (3.1% of all the compounds screened), were fast-acting, i.e., phenotypes were recorded at the 24 h time-point (Table S1).

When the Microsource Spectrum and Killer collections are broken down into their component drug classes (Table 1, Table S3), and using the 7 d confirmatory screen data, the greatest percentage of hits per class, as might be expected, was for the anthelmintics (29%). Within this group the known anti-schistosomals PZQ and hycanthone were identified, as were other anthelmintics such as niclosamide, bithionol and pyrvinium pamoate (Table S3). Examples of other drug classes returning percentage hits greater than 10% are the antibiotics, fungicides, antineoplastics, dopaminergics and seratonergics.

**Table 1:** Phenotypic hits for *S. mansoni* schistosomula as a function of compound class.

Compound Class (Number of compounds per class)	Primary hits (after 7 days)	Confirmatory hits (after 7 days)	Confirmatory hits (after 24 hours)
Adrenergic (40)	2	2	1
Analgesic (35)	0	0	0
Anesthetic (17)	1	0	1
Anthelmintic (24)	7	7	5
Antibiotic (178)	20	18	11
Antihistamine (14)	0	0	0
Antihyperlipidemic (16)	2	2	0
Antihypertensive (25)	1	0	1
Antiinflammatory (48)	1	1	1
Antineoplastic (77)	10	10	4
Antioxidant (12)	0	0	0
Antiprotozoal (31)	4	4	2
Antiviral (11)	1	1	0
Cholinergic (64)	4	3	3
Diuretic (18)	0	0	0
Dopaminergic (38)	7	6	5
Estrogen/progesterone (33)	2	1	1
Fungicide (29)	6	5	3
GABAergic (13)	0	0	0
Glucocorticoid (27)	0	0	0
Herbicide (31)	1	1	0
Insecticide (25)	3	2	1
Muscle relaxant (13)	1	1	1
Seratonergic (38)	7	6	6
Other (480)	11	10	4
Unknown (655)	27	25	11
**TOTAL (1,992)**	**118**	**105**	**61**

See Table S3 for details of the compounds assigned to each drug class.

Upon completion of the first (schistosomular) component of the screen workflow and bearing in mind the target drug profile for schistosomiasis [29], the first of two GO/NO-GO filters was enacted (Figure 1; Table S1). Fast-acting compounds (phenotypes by 24 h) were prioritized and those clearly toxic or otherwise inappropriate for oral use were removed. Accordingly, all but four (phenylmercuric acetate, thimerosal, benzalkonium chloride and homidium bromide) of the 61 fast-acting compounds were prioritized for *in vitro* tests against adult parasites

### Apparent structure activity relationship (SAR) for tricyclic psychoactive drugs inducing an ‘overactive’ phenotype in schistosomula

**Figure 4:**
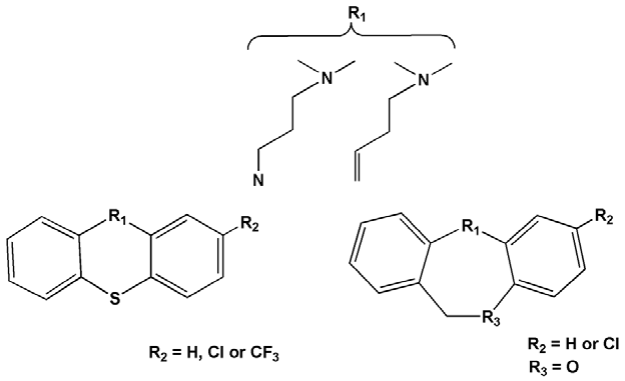
SAR for the phenothiazine and dibenzazepine classes of psychoactive drugs eliciting the ‘overactive’ phenotype in schistosomula. See Table S4 for individual compounds and Supplementary Videos 1 and 2 to compare phenotypes between ‘overactive’ and control worms.

During the first component of the screen workflow it became clear that certain tricyclic psychoactive compounds within the Microsource collections, notably the phenothiazines and dibenzazepines, elicited a striking “overactive” phenotype that lasted for the 7 day duration of the experiment. To verify the result, a mini-screen incorporating the tricyclic psychoactives and structurally related compounds was set up over three logs of concentration; 1.0, 0.1 and 0.01 µM (Table S4). The overactive phenotype was confirmed at 1.0 µM for the same compounds and extended to 0.1 µM, but not 0.01 µM. Examination of structural-activity relationships (SAR) in the phenothiazine and dibenzazepine classes indicated the importance of an unsubstituted propyl side chain possessing a terminal dimethylamine function (Figure 4; Table S4). Even subtle alteration of this pharmacophore, such as the introduction of a branching methyl substituent, led to abrogation of the phenotype (compare promazine and trimeprazine in Table S4). More dramatic modification of the side chain (e.g., shortening, introduction of terminal piperazine or piperidine moieties) similarly abrogated the overactive phenotype. The trend held whether carbon was substituted for nitrogen at position 10 of the phenothiazine (or at position 11 of the dibenzazepine) or whether the tricyclic core was altered internally or substituted.

### Adult screen

Of the 57 compounds passing the first GO/NO GO filter and pursued in the second (adult) component of the screen workflow, 30 were hits (1.5% of the 1,992 compound total) after the maximal screen time of 4 days at 1 µM (Table S2). Seventeen and 10 compounds generated phenotypes by the 7 h and 24 h time points, respectively. Included in this set was the antibiotic, anisomycin, and the anthelmintics PZQ (worms visibly contracted and shrank within seconds of adding 1 µM), niclosamide, pyrvinium pamoate and bithionol. Three compounds, including a former chemotherapy of schistosomiasis, hycanthone, generated phenotypes after 2 days.

**Figure 5:**
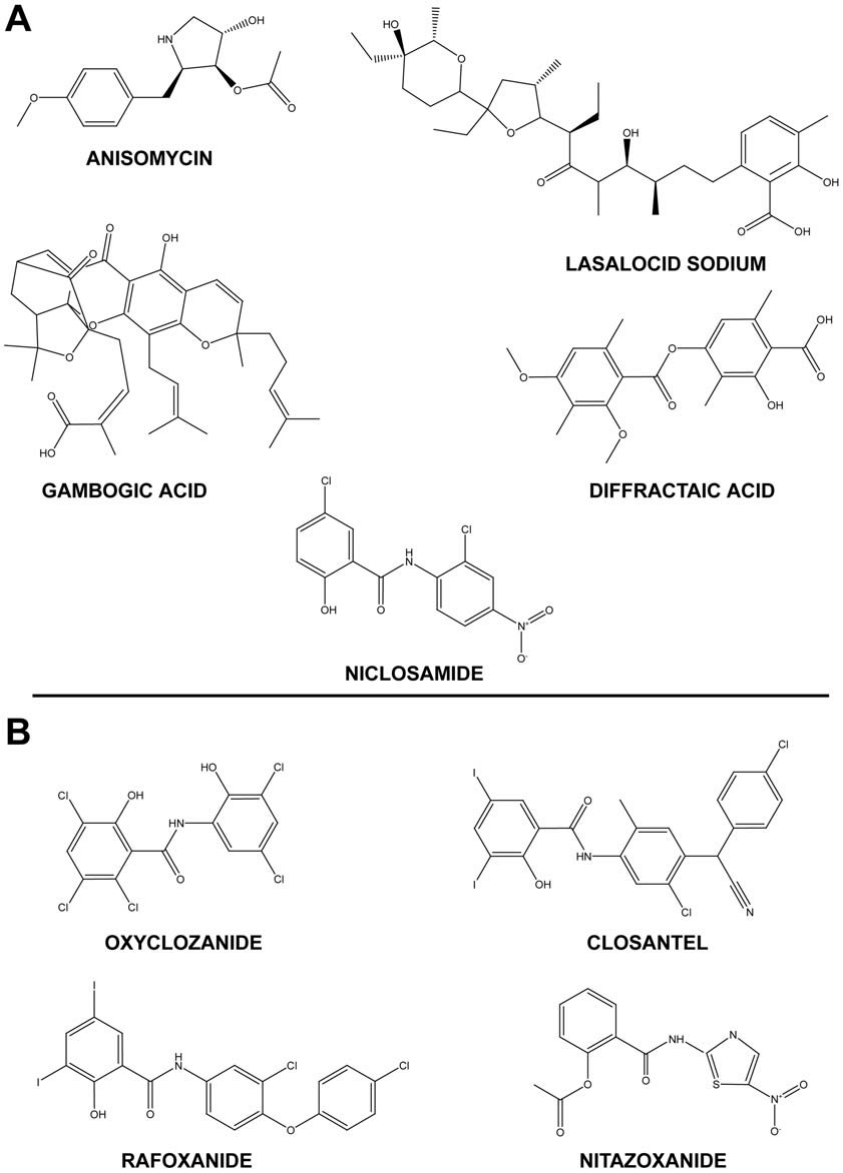
Structures of compounds prioritized for efficacy tests in the murine model of schistosomiasis mansoni. (A) Hit compounds arising from the *in vitro* components of the screen workflow and (B) analogs of niclosamide currently marketed as veterinary anthelmintics or as an intestinal anti-protozoal in humans (nitazoxanide) and tested in the murine model of schistosomiasis.

To prioritize candidates for tests in the murine model of schistosomiasis, a second GO/NO GO filter was implemented utilizing a greater number of considerations than for the first filter (Figure 1; Table S2). As for the first filter, due provision was made for the TPP for schistosomiasis therapy: fast-acting compounds (phenotypes within 7 and 24 h) were prioritized but with a preference now for those that generated the most severe phenotypes, e.g., “teg. bleb” and “dead”. Prioritizations were counter-balanced by available knowledge of compound efficacy, toxicity, and side-effects. Thus, bithionol was deprioritized due to its lack of efficacy in schistosomiasis patients [38] and celastrol due to its toxicity in mice (death within one day of a single 10 mg/kg i.p. dose (R. Swenerton, unpublished data). Likewise, rhodomyrtoxin B displays low LC (lethal concentration)_50_ values of between 2 and 20 µM against Hep-G2 (human hepatocellular carcinoma) and MDA-MB-231 (human mammary adenocarcinoma) cell lines [39] and was, therefore, not considered further. The tricyclic psychoactive compounds (e.g., chlorpromazine, imipramine) were also deprioritized at this stage given that the less severe ‘overactive’ phenotype that appeared by 2 h was either transient (lasting only until the 24 h time point) or without progression to a more severe phenotype(s). Also, their psychoactivity and associated side effects, e.g., sedation, detracted from their immediate consideration. In all, therefore, five compounds were prioritized for efficacy tests in the model of murine schistosomiasis: two antibiotics, anisomycin and lasalocid sodium; two natural products, diffractaic acid and gambogic acid; and the helminthicide/molluscicide, niclosamide (Figure 5A, Table S2).

### Murine model

For the third and final component of the screen workflow, all compounds were administered orally in 2.5% Cremophor EL (unless otherwise stated) either once (QD) and/or twice daily (BID) for 4 days to mice with patent *S. mansoni* infections (42 days p.i.).

**Figure 6:**
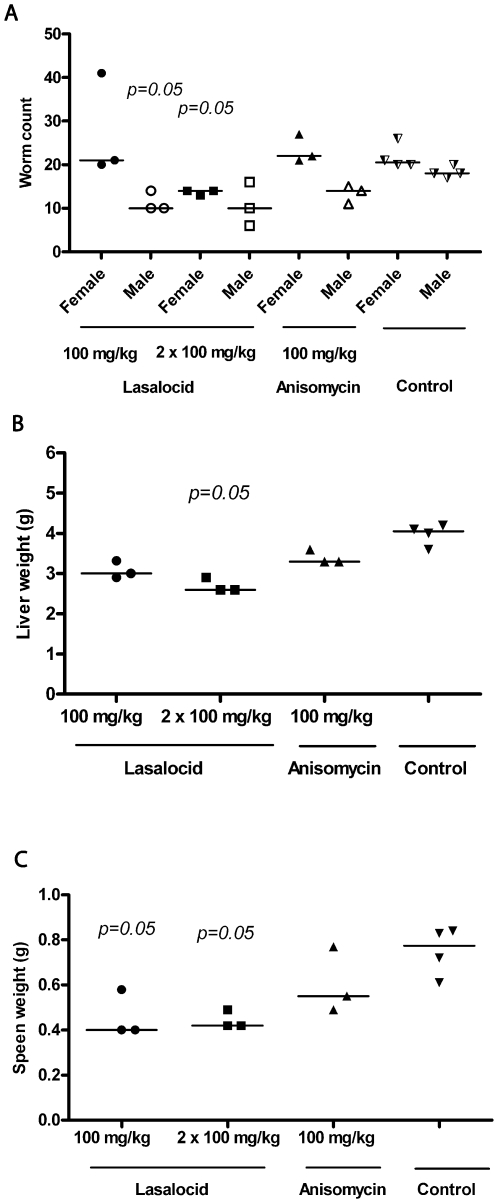
Effect of lasalocid sodium and anisomycin on male and female worm burdens, and organ pathology (as measured by weight) in mice infected with *S. mansoni*. Compounds were administered orally QD or BID at the doses indicated for 4 days 42 days after infection with 140 *S. mansoni* cercariae. Points represent data from individual treated or untreated (control) infected mice. The horizontal bars represent median values. Significance (*p*) values are indicated where *p*≤0.05. Egg burdens were calculated as single values per treatment group (see text for details).

The antibiotic, anisomycin, at 100 mg/kg p.o. QD, had no significant effect on male or female worm burdens (Figure 6A), yet decreased hepatic egg burdens by 36% (calculated as an averaged single value for the treatment group). Neither liver nor spleen weights were significantly different from those of the untreated control group (Figure 6B and C). Increasing to a BID administration resulted in toxicity; all mice died between 3 and 10 days after the commencement of treatment. The ionophoric antibiotic, lasalocid sodium, was better tolerated by mice. Significant decreases in male (44%) and female (41%) worm counts were measured at 100 mg/kg QD and BID, respectively (Figure 6A). For egg burdens, reductions of 39 and 55% (calculated as averaged single values per treatment group) were measured QD and BID, respectively. Lasalocid sodium also significantly improved organ pathology compared to controls (Figure 6B and C).

For the *Usnea* lichen metabolite, diffractaic acid poor solubility in aqueous media or vehicle prevented the ability to accurately gavage mice. Therefore, i.p. administration in 50 µL 100% DMSO over a range of doses (10, 40 and 100 mg/kg) was performed to determine compound efficacy while also observing for overt toxicity. No decrease in worm or egg burdens was measured at the lower doses, whereas at 100 mg/kg, all mice died within 7 days of the cessation of treatment (data not shown). Gambogic acid, a xanthone isolated from various species of the *Garcinia* tree, was recently shown to be non-toxic in rats after oral administration every other day for 13 weeks at 30 and 60 mg/kg in 2% carboxymethylcellulose-sodium [40]. Using the same vehicle at 100 mg/kg QD, no effects on worm or egg burdens were recorded (data not shown).

The final compound, niclosamide (2′5-dichloro-4′-nitrosalicylanilide), a molluscicide and intestinal helminthicide, is poorly absorbed across the intestinal wall. Therefore, we obtained from Bayer a wettable powder formulation of the compound (marketed as Bayluscide WP 70) that is better absorbed (in rats about a third of an oral dose [41]). However, in tests with both niclosamide formulations at 100 mg/kg BID, no effects on worm or egg burdens were noted (data not shown). Further, niclosamide was ineffective at 100 mg/kg BID in two additional vehicles (2% Tween80/7% ethanol and 6% PEG 4000/2%Tween 80/7% ethanol). In a final attempt to demonstrate efficacy, i.p. administration of niclosamide at 100 mg/kg BID in 100 µL 25% DMSO was without effect – upon dissection of mice the compound was noted to adhere as a solid mass at the injection site on the inner side of peritoneal membrane, indicating that much of the compound had not been absorbed.

### Screening niclosamide analogs in the murine model

Based on the strong *in vitro* efficacy measured for niclosamide against both schistosomula (Table S1) and adults (Table S2), and notwithstanding its lack of *in vivo* efficacy, we searched for structurally related compounds that are commercially available and have demonstrated oral efficacy against helminths or protozoa. Three salicylanilides, closantel, oxyclozanide, and rafoxanide, and the nitrothiazolyl-salicylamide, nitazoxanide (Figure 5B) were purchased. The salicylanilides are well-established drugs used in the agribusiness sector as helminthicides, including against liver fluke disease caused by *Fasciola hepatica* [42]. They have also displayed variable efficacies in experiments with farm animals harboring agriculturally important Indian schistosome species such as *Schistosoma incognitum* and *Schistosoma nasale* ([43],[44]). Nitazoxanide (marketed as Alinia) is approved for the treatment of diarrhea caused by *Cryptosporidium parvum* and *Giardia lamblia* and has shown efficacy against human fascioliasis hepatica [45]. Accordingly, we judged there to be sufficient precedent and data available to move these compounds straight into our mouse disease model. The oral efficacy of these drugs were compared to the ‘gold standard’ drug, PZQ.

**Figure 7:**
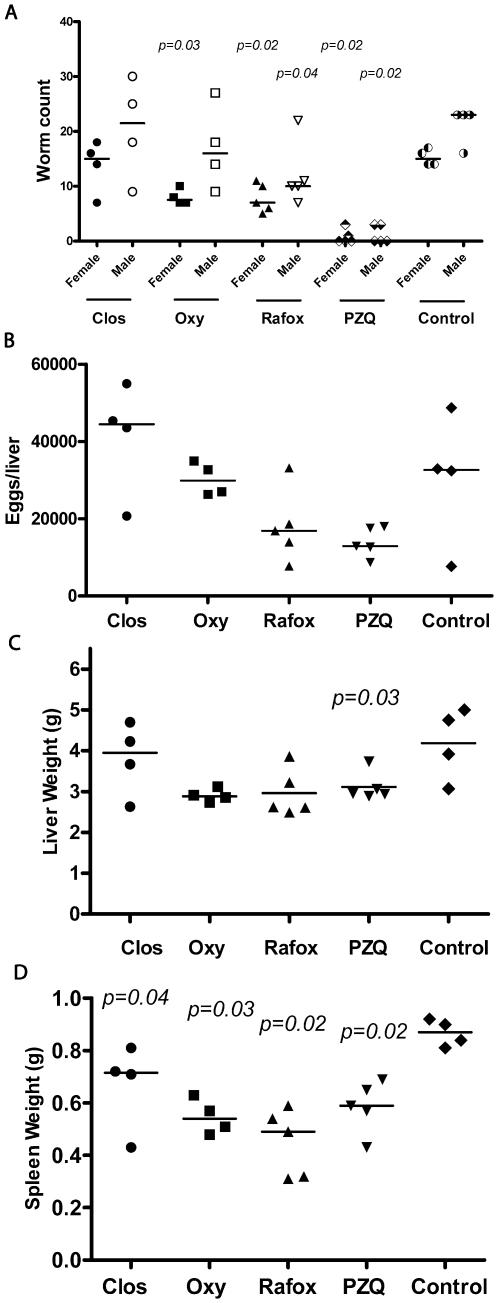
Effect of closantel, oxyclozanide, rafoxanide and PZQ on male and female worm burdens, egg burdens and organ pathology in mice infected with *S. mansoni*. Compounds were administered orally for 4 days 42 days after infection with 140 *S. mansoni* cercariae. Doses administered were: 100 mg/kg BID for closantel and oxyclozanide, 50 mg/kg QD for rafoxanide and 100 mg/kg QD for PZQ. Points represent data from individual treated or untreated (control) infected mice. The horizontal bars represent median values. Significance (*p*) values are indicated where *p*≤0.05.

**Figure 8:**
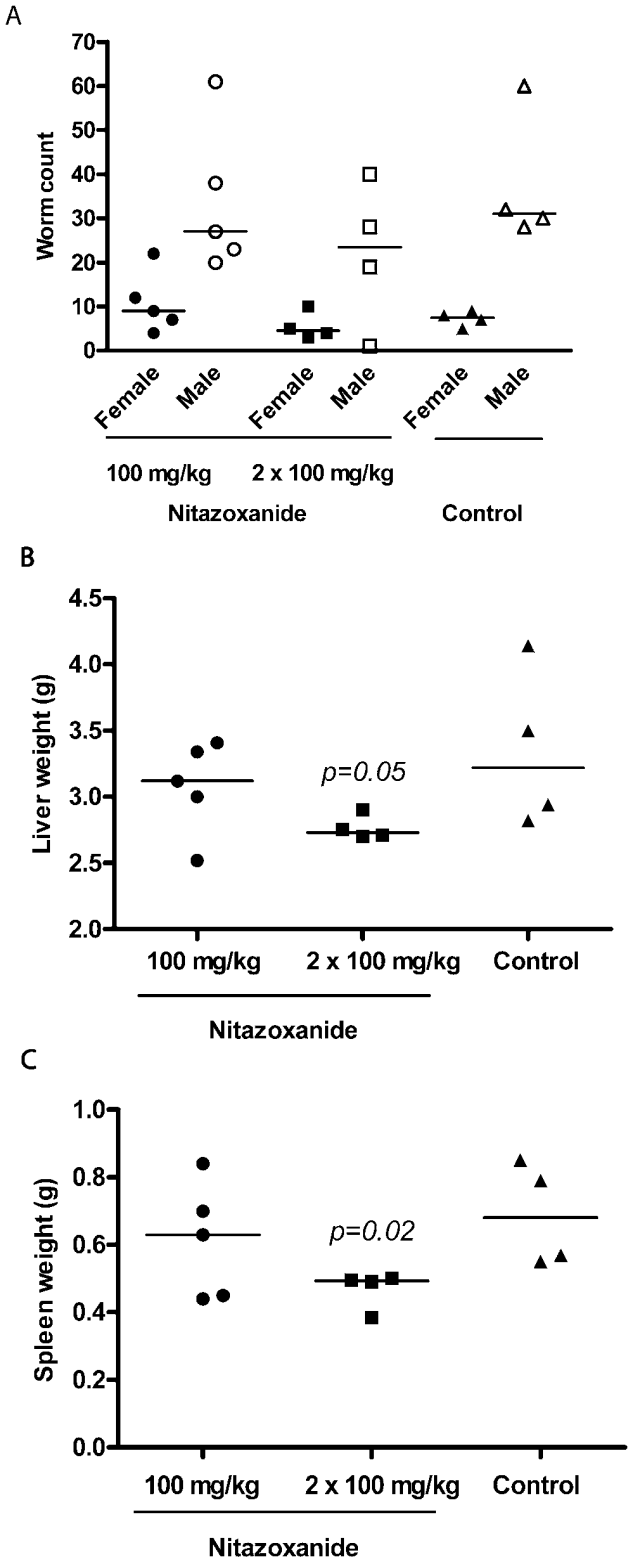
Effect of nitazoxanide on male and female worm burdens, and organ pathology in mice infected with *S. mansoni*. Compound was administered orally at the doses indicated for 4 days 42 days after infection with 140 *S. mansoni* cercariae. Points represent data from individual treated or untreated (control) infected mice. The horizontal bars represent median values. Significance (*p*) values are indicated where *p*≤0.05. Egg burdens were calculated as single values per treatment group (see text for details).

When administered at 100 mg/kg p.o. QD for 4 days commencing at 42 days p.i., PZQ significantly decreased male (91%) and female (87%) worm burdens (Figure 7A) and these were associated with a decreased hepatic egg load (60%; Figure 7B) and improved organ pathology (Figure 7C and D). The decrease in egg burden was not considered significant, however, due to the low load recorded for one of the control mice. The few worms surviving treatment and recovered by perfusion were the smallest seen in all of the *in vivo* experiments and some were physically damaged (not shown). By comparison, BID administration of the salicylanilides, closantel and oxyclozanide, at 100 mg/kg yielded less pronounced effects on worm burdens (only oxyclozanide significantly decreased female loads by 53%; Figure 7A) and egg burdens were not affected (Figure 7B). However, worms recovered after oxyclozanide treatment were smaller than controls (not shown) and organ pathology (significantly so for the spleen) was also improved (Figure 7C and D). The third salicylanilide tested, rafoxanide, at either 100 mg/kg QD or BID, caused mouse mortality within 5 days of the cessation of treatment, however, this seemed not to be due to systemic toxicity *per se* but rather an accretion of drug in the stomach that caused gastric blockage. At 50 mg/kg QD (i.e., half the dose of PZQ) all mice survived. The drug was the most effective of the niclosamide analogs tested significantly decreasing male (56%) and female (50%) worm loads (Figure 7A). Also, worms recovered were smaller than controls (not shown). Egg counts were decreased by 49%, but as noted for PZQ above, the value was not significant due to an outlier control mouse with a particularly low hepatic egg count. Rafoxanide was as effective as PZQ in improving organ pathology (Figure 7C and D). The final niclosamide analog tested, nitazoxanide, was without effect on worm burdens at 100 mg/kg QD and BID (Figure 8A) but significantly improved organ pathology BID (Figure 8B and C). Nitazoxanide also decreased egg outputs by 34% (calculated as a single averaged value per treatment group).

## Discussion

Compared to the high profile activity supporting the development of novel anti-protozoal and anti-infective therapies [7],[8] the pace of drug discovery for anti-schistosomals (and anthelmintics in general) is slow. For schistosomiasis, a number of mutually suppressive factors are responsible. Perhaps foremost is the success and clinical reliability of PZQ that have dampened investment in a dedicated drug development pipeline, a situation in stark contrast to malaria and protozoal NTDs for which drug toxicity and/or increasing drug resistance fuel a number of multinational PPP programs to identify new therapeutics [7]. Other contributing factors are; the relatively small number of groups involved in anti-schistosomal discovery, the need to maintain a complex life-cycle that generates finite parasite yields and the long identification and development time lines associated with phenotypic (whole organism) screens as traditionally prosecuted in animal models and/or *in vitro* with adult worms. Despite these drawbacks, phenotypic screening has successfully identified PZQ and other vital anthelmintics (e.g., albendazole and ivermectin) that are in medical use today. Most often, the compounds originated in the animal health sector as part of its discovery programs to identify veterinary anti-parasitics [46],[47].

For this report, we have designed a phenotypic screen process by introducing a three-component workflow (Figure 1) that places *S. mansoni* schistosomula at its apex. The intent is to streamline and accelerate the identification of anti-schistosomal compounds by interfacing the helminth with the microtiter plate (96- and/or 384-well) formatted compound libraries and associated robotic liquid handling systems now standard in industry and many academic institutions, and routinely employed to screen the more tractable protozoan parasites [7]. Given their small size (∼200×60 µm) schistosomula are readily adaptable to the 96-well plate format and survive for 7 days with less than 10% mortality under the conditions described. Also, they are quickly and easily transformed from the invasive cercariae that are harvestable in their tens of thousands on at least a weekly basis from vector snails. Both points are immediately attractive and conducive to designing a higher throughput screen workflow. The alternative adult parasite is too large for 96-well plate formatting and can only be harvested from vertebrate hosts (e.g., mice and hamsters) in more limiting numbers, and entails considerable expenditure associated with animal procurement and maintenance. That stated, adults are not omitted entirely from the screening process but are placed downstream of schistosomula when the number of compounds to be tested is more manageable – a consideration also of importance for the final component of the workflow involving the animal model of schistosomiasis.

The Microsource collections of 2,160 compounds were prosecuted at a throughput of 640 compounds/month for the primary schistosomular screen component. With one full-time technician and an associate analyst it took 20 weeks to complete the *in vitro* screening of the collections against schistosomula and adults. Efforts to at least double the screening capacity of the first component of the screen workflow are being studied, for example, through the employment of additional staff and expansion of our in-house *S. mansoni* life-cycle. Also, screen formatting to 384-well plates is being considered together with the complete automation of both the liquid handling of the parasite and phenotype identification and categorization. Data accrued from each component of the screen workflow is available as a flat file online at the UCSF Sandler Center's ‘Low Hanging Fruit’ website http://www.sandler.ucsf.edu/fruit.html and at http://www.collaborativedrug.com/, a database that can be mined across compounds and parasites to identify molecules and chemistries of interest. Both sites are continually updated as screening campaigns are concluded and it is hoped that the data will contribute to drug discovery efforts for schistosomiasis and other NTDs.

The descriptive approach employed here to annotating the dynamic responses of this metazoan parasite to chemical stimuli differs necessarily from the single end-point fluorometric or colorimetric assays, now routine for high-throughput assays of single-celled organisms and with which a rigorous quantification of a live *versus* death ratio is relatively facile [7]. Given the traditionally slower compound throughput for schistosome screening, the demand for marker dyes or reagent-based kits has simply not been present with visual-based scoring systems being the norm [21],[22],[23]. Our attempts to incorporate nuclear dyes (e.g., propidium iodide and DAPI) as a quantitative marker of cell death in schistosomula did not correlate with the clear deleterious action of some compounds observed under bright field microscopy (Caffrey, unpublished data). Often dyes were simply excluded from crossing the schistosome tegument regardless of worm condition. Thus, our decision to visually classify phenotypes, though potentially prone to subjectivity, turned out to be a consistent semi-quantitative approach as employed, i.e., using blind consensus determination of bioactivity by trained analysts familiar with the parasite's phenotypic manifestations (see discussion below). Further, it might be argued that the workflow, because of its simplicity, and without the need for expensive kits or reagents, is more adaptable to a greater variety of discovery settings. Nevertheless, we are aware that any attempt to improve the quantitative rigor of hit identification and classification should be a primary goal. Accordingly, we are examining a number of automated time-lapse image capture platforms to improve efficiency and accuracy, including the ability to record phenotypes too subtle to be observed with the human eye.

In addition to increased throughput and improved automation, the logistical decision to commence the screen workflow with the schistosomulum stage has both potentially advantageous and disadvantageous consequences. Of advantage is that the workflow may identify compounds that are active against both immature and adult stages of parasite, or, at least, against immature parasites. This is important in the context that the current chemotherapy, PZQ, is markedly less effective against the immature (migratory and sub-adult) parasite compared to mature egg-laying adults [10],[11],[48]. Thus, the identification and development of a small molecule prophylaxis for individuals harboring immature parasites, such as in areas of higher transmission, would be of considerable value. By extension, the opportunity to develop a combination (possibly synergistic) therapy with PZQ to decrease the threat of resistance to the latter may also be facilitated by the present screen workflow that commences with the schistosomular stage rather than adults. The concept of a PZQ-based combination therapy based on reciprocal drug efficacy against immature and mature parasites has already shown value with the artemisinin class of compounds ([14] and references therein).

A possible disadvantage of the current screening approach is the potential for missing compounds that are inactive against schistosomula, yet, nevertheless, might have yielded interesting phenotypes against adults worms. We accept this possibility as part of the overall goal to streamline and accelerate the identification of anti-schistosomal compounds. We would emphasize that, where smaller compound collections are concerned, the screen workflow can be conducted in a non-hierarchical manner whereby every compound is tested against both schistosomula and adults. Whether a compound is a hit or not or whether it passes or fails the GO/NO GO criteria as implemented here, all screen data are made publicly available for (re)interpretation.

As to the choice of compound collections maintained at the UCSF SMDC (http://smdc.ucsf.edu/) to initiate the screen workflow, the Microsource Spectrum and Killer collections seemed appropriate for a number of reasons. First, the collections comprise a tractable set of 1,992 unique compounds, so that with a modest throughput the first and second components of the workflow were complete within 20 weeks. Secondly, the collections have a track record of yielding novel leads against other parasites including *Plasmodium falciparum* [31],[33] and *Trypanosoma brucei* [32]. Finally, the collections contain a chemically diverse set of natural and synthetic small molecules, 41% (821 compounds) of which are drugs already FDA-approved. From a drug-repositioning standpoint, this is particularly attractive because of the existence of clinical data (e.g., adsorption, distribution, metabolism, excretion and toxicity (ADMET)) that could contribute to fast-tracking these compounds as anti-schistosomals, especially as the compounds are off-patent and without intellectual property concerns.

Of the 118 compounds identified as hits and phenotypically classified after 7 days of incubation in the primary schistosomular screen component, 105 were confirmed. Likewise, for the adult component of the workflow, repeated tests with compounds resulted, in most cases, in the same phenotypes. Thus, our blinded consensus approach to visually recording bioactivity provided reasonable reproducibility. In further support of the strategy, known schistosomicides, including PZQ and hycanthone, were, without fail, identified and consistently characterized, as were other anthelmintics, such as bithionol and niclosamide. Importantly, direct visual observation allowed us to identify and record the multiple and changing phenotypes that are possible with schistosomes and, not least, discover an apparent SAR for tricyclic psychoactive compounds primarily focused on the structure of the side chain. As yet the molecular target(s) of the dibenzazepine and phenothiazine drugs in question is unknown. It is possible that the ‘overactive’ phenotype is not neuroreceptor-mediated but perhaps a result of membrane interference (depolarization?). Nonetheless, it is interesting that, over a two log-fold concentration, compounds designed to interact with different ligand-gated receptors in humans nevertheless yield the same phenotype in the parasite, suggesting that a single parasite receptor or a discrete subset of receptors may be the target. By mining the available genome sequence information for *S. mansoni* [18],[19], one might envisage RNA interference of candidate cholinergic, dopaminergic or seratonergic receptors in an effort to modulate the overactive phenotype. This would prove the hypothesis that these compounds share a receptor and aid the development of an SAR-based drug discovery program.

Both GO/NO GO filters in the workflow were designed in consideration of the TPP demanded for new anti-schistosomal drugs that employs the current therapeutic, PZQ, as a gold-standard. The bar is high - PZQ decreases worm burdens by between 60 and 90% in a single oral dose [3],[48]. Criteria of speed of appearance, phenotype severity (death preferred) and oral suitability were balanced with clinical data on dosage and safety. As interpreted for this report, the second GO/NO GO filter removed a number of compounds and compound classes that elicited striking phenotypic effects. Among these were the tricyclic psychoactive compounds. The ‘overactivity’ they elicited may yet prove therapeutically significant, perhaps by disrupting the parasite's migratory program or its ability to remain in position within the host. Targeting neurotransmission (if that indeed is the mechanism in schistosomes) is a successful chemotherapeutic strategy for other helminths ([49]). We will examine the efficacy of these compounds in the murine disease model at low doses either alone or in combination with PZQ.

As implemented, the second GO/NO GO filter prioritized five (niclosamide, anisomycin and lasalocid sodium, diffractaic acid and gambogic acid) of the 30 hit compounds identified in the second (adult) component of the screen workflow for tests in the animal model of schistosomiasis. All five quickly kill (within hours) both schistosomula and adults at 1 µM in culture. Also, LD_50_ toxicity data are available against which a dosing regimen can be prepared and four of the five top hits (excepting diffractaic acid) can be administered orally.

In the murine model of disease, anisomycin and lasalocid sodium demonstrated varying parasitological efficacies and amelioration of hepatic and splenic pathology. Though not as effective as PZQ, we consider the identification of these novel *in vivo* anti-schistosomal activities as proof that the screen as conceptualized (w.r.t. drug-repositioning) and implemented can identify potentially interesting and chemically diverse compounds. Such compounds might be employed for therapy as is or as leads for further derivatization (e.g., anisomycin is chemically relatively simple) in order to improve bioactivity while reducing toxicity. The point is further underscored with the niclosamide analogs. Though niclosamide and its wettable powder formulation were ineffective in the mouse model of disease, niclosamide's rapid and severe *in vitro* bioactivity encouraged us to search for other salicylanilide analogs. We identified a number that are well-established in the veterinary sector and possess better oral bioavailability with systemic anthelmintic activity, including against related trematode parasites [42]. The often significant *in vivo* efficacy of these drugs in both the parasitological and pathological parameters measured, particularly with rafoxanide, encourage further study of the salicylanilides as a source of anti-schistosomal leads. These investigations are underway.

In conclusion, as a central component in a pre-clinical drug discovery pipeline for schistosomiasis, we have developed a partially automated phenotypic screen workflow of increased throughput. All data arising are posted and updated online and work continues to improve automation, rigor and throughput. As currently performed, the workflow has already identified a diversity of hit compounds and chemistries *in vitro*, as well as lead compounds orally bioactive in a short time frame commensurate with the TPP for chemotherapy of schistosomiasis [29]. Critically, the drug-repositioning dimension with the availability of clinical data for many of these hits and leads can be leveraged to optimize further compound development. Accordingly, screens of other libraries containing known drugs are ongoing. Given the possibility of the emergence of resistance to the current PZQ monotherapy, and because any strategic planning for therapy of infectious diseases should incorporate provisions for drug combinations, our future studies will focus on the *in vivo* performance of the present and future lead compounds, either alone or in combination, including with PZQ.

## Supporting Information

Table S1 Phenotypes recorded upon primary and confirmatory screening of the Micosource ‘Spectrum’ and ‘Killer’ Collections at 1 µM in vitro against *S. mansoni* schistosomula.
(3.31 MB XLS)

Table S2 Phenotypes recorded against *S. mansoni* adults at 1 µM in vitro with those hit compounds that arose in screens against schistosomula.
(0.05 MB XLS)

Table S3 Compounds yielding phenotypes in *S. mansoni* schistosomula as a function of drug class.
(0.40 MB XLS)

Table S4 Apparent structure activity relationship for tricyclic pyschoactive compounds (e.g., dibenzazapines and phenothiazines) within the Microsource collections that generate the ‘overactive’ phenotype in *S. mansoni* schistosomula at 1.0 and 0.1 µM.
(0.11 MB XLS)

Video S1 *S. mansoni* schistosomula demonstrating the ‘overactive’ phenotype after 7 d in vitro in the presence of 1 µM imipramine.
(6.53 MB MOV)

Video S2 Control *S. mansoni* schistosomula incubated for 7 d in vitro.
(7.04 MB MOV)
